# R-loops: formation, function, and relevance to cell stress

**DOI:** 10.15698/cst2019.02.175

**Published:** 2019-01-21

**Authors:** David F Allison, Gang Greg Wang

**Affiliations:** 1 Lineberger Comprehensive Cancer Center, University of North Carolina at Chapel Hill School of Medicine, Chapel Hill, NC 27599, USA.; 2 Department of Biochemistry and Biophysics, University of North Carolina at Chapel Hill School of Medicine, Chapel Hill, NC 27599, USA.

**Keywords:** R-loops, RNA-DNA hybrid, replication fork stalling, gene transcription, genome instability, mRNA splicing, DNA damage, chromatin modification, cancer, ataxia

## Abstract

Exposure of genomic, single-stranded DNA (ssDNA) during transcription and replication creates opportunities for the formation of inappropriate secondary structures. Cells manage this exposure by using topoisomerases and helicases to reduce the inherent topological stress that arises from unwinding the double helix and by coating ssDNA with protective protein complexes. Interestingly, specific DNA-RNA hybrids, known as R-loops, form during transcription and exist in homeostasis throughout the genomes of prokaryotes and eukaryotes. These hybrids nucleate from guanine rich clusters in the template strand and extend across GC rich spans of transcribed genes. *In vivo* regulatory functions have evolved from R-loops, including regulation of gene expression and telomere lengthening. However, they also exist as a form of stress, particularly when replication forks collide with the transcription machinery. New methodologies and models are being developed to delineate the biology of R-loops, including those related to cell stress-based diseases like cancer. As accumulation of R-loops is associated with disease, targeting molecular pathways that regulate their formation or removal could provide new avenues for therapeutic intervention. This review covers recent understandings of the molecular basis for R-loop formation, removal, and biological outcomes in the context of cellular stress.

## INTRODUCTION

**Figure 1:**
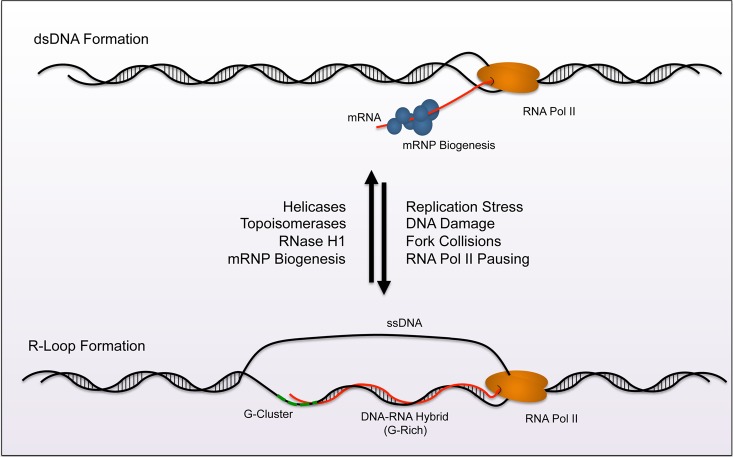
FIGURE 1: Model of R-loop homeostasis. A homeostasis exists between formation and removal of R-loops across the genome. Normally, mRNP biogenesis machinery targets nascent RNA and processes and prepares the strand for nuclear export (top). Topoisomerases and Helicases aid in preventing R-loop formation by reducing exposure times of ssDNA during transciption. RNase H removes R-loops by specifically digesting RNA in helical formation. Together these processes reduce R-loop accumulation during transcription. Factors that lead to pausing of RNA polymerase render conditions favorable for R-loop formation (replication stress, DNA damage, fork collisions, etc). R-loops nucleate from G-clusters (dashed green line). The loop then extends along GC rich sequences within the gene during elongation (bottom). The balance between removal and formation creates an equilibrium at nucleation sites across the genome.

A variety of topological, structural and hybridization events occur during DNA replication and gene transcription. Unwinding of the DNA double helix provides access for polymerase to a template strand, and creates torsional stress that can manifest anomalous formation of “non-traditional” moieties. One such structure, known as an R-loop [1, 2], occurs during transcription (**Figure 1**). As RNA polymerase progresses along the DNA double helix, newly transcribed RNA threads back to hybridize with the transiently accessible template strand and displace the non-template strand. Structurally, the hybrid adopts an intermediate conformation between B-form double-stranded (ds)DNA and Aform dsRNA. This form carries more stability than dsDNA and must be enzymatically resolved in order to restore the native double helix. A homeostasis emerges from the constant formation and removal of R-loops throughout the genome. Moreover, their presence has biological relevance in regulating gene expression and specialized rearrangement events. Misregulation of R-loop homeostasis promotes genomic instability is associated with progressive neurodegenerative disorders or cancer. The purpose of this review is to detail the current understanding of R-loop contribution to cell stress and disease.

## FORMATION AND REMOVAL OF R-LOOPS

R-loops canonically nucleate from guanine-rich clusters (G-clusters) during gene transcription [3, 4], and these clusters increase the favorability of nascent RNA to anneal with complementary single-stranded (ss)DNA (**Figure 1**). Research demonstrates that RNA containing four or more consecutive guanine residues near the 5' end possesses significantly higher rates of R-loop formation due to this nucleation requirement. After initiation of the R-loop, RNA-DNA hybridization is elongated and stabilized by a subsequent stretch of guanine rich (G-Rich) sequence. Elongation becomes less favorable when G-rich content decreases and the structure terminates. Other factors also promote R-loop formation and reduce the requirement for G-Rich sequence. For example, increased negative supercoiling on the trailing fork of the transcription bubble extends the opportunity for potential interactions between newly transcribed RNA and the template strand (reviewed in [5]). Additionally, nicks in the non-template strand can promote DNA-RNA hybridization of nascent RNA to the template strand, even when the G-Rich sequence is distal to the initiating G-cluster [3, 4].

The build-up of R-loops is counteracted through a variety of mechanisms that maintain transcription bubble integrity, including nuclease activity and resolution of topological stress (**Figure 1**). These mechanisms are detailed in recent review articles [5-9]. Briefly, distinct enzymes carry out complementary actions to prevent accumulation of R-loops. RNase H1 and RNase H2 utilize 5'–3' exonuclease activity to remove RNA from the loop. These enzymes are evolutionarily conserved in prokaryotes and eukaryotes and are the only RNA-specific ribonucleases known to digest hybridized RNA. Complementary to RNase H1/2, cells also possess helicases that “untangle” DNA-RNA lesions like mammalian DHX9 and aquarius (AQR) [10, 11]. Other helicases known to resolve R-loop structures include the yeast Sen1p orthologue, senataxin (SETX) [12] and an ATP-dependent DNA helicase PIF1 [13].

Preventative mechanisms exist as an additional measure of protection against R-loop accumulation (**Figure 1**). Topoisomerases work to decrease negative supercoiling that increase access of nascent mRNA to G-clusters on the template DNA. Topoisomerase 1 (TOP1) and Topoisomerase 2 (TOP2) have both been shown to relieve torsional stress and prevent R-loop accumulation at the rDNA locus [14]. Moreover, topoisomerase 3B (TOP3B) reduces R-loop formation at highly expressed genes like the protooncogene MYC [15]. Biogenesis of messenger ribonucleo-protein particles (mRNP) also prevents R-loop accumulation. During mRNP biogenesis, RNA-binding proteins (RBP) interact with nascent mRNA to promote mRNA processing and export from the nucleus. In eukaryotic cells, the THO/TREX complex, as well as Serine/Arginine Rich Splicing Factor (SRSF1), binds ssRNA to initiate mRNP maturation, and these interactions prohibit R-loop formation [16-18]. Additional mRNA splicing and surveillance machinery such as the exon junction complex (EJC) and the exosome also reduce R-loop accumulation by sterically prohibiting interactions between the nascent mRNA and exposed ssDNA [18, 19].

Epigenetic mechansims act upstream of topoisomerase and helicase function in R-loop homeostasis. For example, the MYST family acetyltransferase MOF aids in preventing R-loop accumulation that arises from replicative stress. Depletion of MOF decreases replication fork (RF) speed and potentiates fork stalling, factors that contribute to R-loop nucleation. Furthermore, recruitment of the repair machinery to sites of DNA damage occurs through MOF-mediated ubiquitination [20], while an interaction between THO and SIN3A has been described where histone deacetylation prevents R-loop formation [21]. How acetylation regulates R-loop formation is likely context dependent and further studies should delineate these differences.

Collectively, the cooperative activities of transcription bubble maintenance and mRNA processing machinery resolve and prevent R-loop accumulation. Each individual component contributes to the homeostasis of R-loops, and their respective regulation determines the local prevalence of these hybrid structures throughout the genome.

## TECHNIQUES DEVELOPTED TO STUDY R-LOOPS

Since being first described in 1976 [2], several techniques have been developed to study eukaryotic R-loop biology *in vitro* and *in vivo*. Various plasmid-based systems have been created to study the regulation of R-loop formation and removal. Known R-loop sequences are inserted into plasmids in order to examine the effect of these structures on replication or transcription *in vitro*.

Classically, a series of electrophoresis techniques have been developed to identify sequences that favorably form R-loops *in vitro*. The R-loop structure migrates more slowly in agarose gel electrophoresis and can be visualized with ethidium bromide (EtBr) after *in vitro* transcription [22, 23]. Moreover, the shifted band shows resistance to digestion by RNase A and sensitivity to digestion by RNase H1. These exonucleases target ssRNA and helical RNA, respectively. Using labeled uridine during the *in vitro* transcription step increases sensitivity of the assay. Moreover, it eliminates signal from dsDNA that appears when using a general nucleic acid stain like EtBr. As a method to identify perturbations in the dsDNA helix, bisulfite modification can be used to detect R-loops [22]. Briefly, unpaired cytosines on the displaced anti-sense ssDNA are converted to uracil through deamination by addition of bisulfite. The uracil subsequently converts CG basepairs to AT basepairs during PCR amplification and the change can be observed by sequencing. Because this technique targets accessible strands of ssDNA, R-loop formation should be verified through additional methodologies.

A powerful, genome-wide technique, known as DNA-RNA immunoprecipitation followed by sequencing (DRIP-seq), uses the S9.6 antibody to specifically pull down DNA-RNA hybrids that are subsequently identified by high throughput sequencing methods to map these structures to the genome [24-27]. While the use of DRIP-seq and its derivatives give an overall picture of R-loop distribution across the genome, called peaks should be validated as some inherent bias has been reported [28]. An alternative high throughput technique known as DNA-RNA *in vitro* enhancement followed by sequencing (DRIVE-seq) utilizes an epitope-tagged, catalytically dead RNase H1 and affinity pulldown to recover DNA-RNA hybrids for sequencing [29, 30]. Depending on experimental conditions, high throughput strategies identify between 1,000 and 20,000 R-loops across the genome. Interestingly, R-loops are largely mapped to gene promoters and terminator regions in human cell lines, showing their potential involvement in regulating RNA pol II-mediated transcription. However, several specialized regions also show enrichment for R-loops where they may act as a more structural component to the genome, including telomeres, ribosomal DNA (rDNA), and transposable elements.

Recently, IP-mass spectrometry techniques were used to identify R-loop interacting proteins in a high throughput format. The study identified the RNA helicase DHX9 and characterized its role in suppressing R-loop accumulation, reducing DNA damage, and promoting transcriptional termination [31]. R-loops can also be detected with microscopy to visually confirm their formation using *in vitro* transcription. For example, the S9.6 antibody, as well as a GFP-tagged DNA-RNA hybrid binding domain of RNase H1, allow for visualization of R-loop foci with fluorescence microscopy [26, 32] or fluorescence *in situ* hybridization (FISH) based techniques [33-35]. Electron microscopy provides another mechanism to study and visualize R-loop structures [36].

The continued development of techniques and methodologies to study R-loop dynamics and the downstream consequences of their accumulation will further detail the contribution that these structures have in gene regulation and genomic instability.

## *IN VIVO* ROLES FOR R-LOOPS IN EUKARYOTIC CELLS

Predictably, formation of a DNA-RNA hybrid creates structural lesions in the genome that can affect replication, transcription, and recombination. Ongoing research continues to detail the *in vivo* role of R-loops as regulatory elements of specialized events in the eukaryotic cell. Previously, mitochondrial origins of replication contain R-loops that are used to facilitate replication of the mitochondrial DNA (mtDNA), a process likely conserved from prokaryotic ancestors [37]. Studies in activated B-lymphocytes also show that R-loops are used at the switch (S) region during immunoglobulin class switching recombination (CSR) to promote deaminization of cytosine and generate a double-strand break (DSB) [22, 38]. In this case, activation-induced deaminase (AID) gains access to the WRC motifs (W=A/T, R=A/G) within the repetitive S regions in the displaced, non-template strand downstream of the IgG promoter [39]. Recently, researchers describe a mechanism where the RNA helicase DDX1 resolves G-quadruplex structures into R-loops at the S region of immunoglobulin heavy chain locus (IgH) in order to recruit AID for CSR [40].

While R-loops contribute to specialized events or structures in the human genome, they also have distinct roles in regulating transcription. In one instance, R-loops regulate epigenetic mechanisms of gene repression. When R-loops form in downstream CpG island promoter regions, they inhibit the activity of DNA methyltransferases to promote expression [26]. In a separate study, R-loop formation can facilitate binding of transcription factors to regulatory elements on DNA. Invasion of anti-sense non-coding RNA (ncRNA) into the promoter region of the vimentin (VIM) gene reduces local nucleosome density. The formed R-loop subsequently increases gene expression by providing access of nuclear factor kappa B (NF-κB) to its binding element [41]. Interestingly, this mechanism runs counter to previously described repressive activities of non-coding (nc)RNA, and provides new insight into how these moeities can positively regulate gene expression.

R-loop formation in G-Rich sequences downstream of the 3' polyadenylation (polyA) signal can promote RNA Pol II pausing [42]. The R-loop structure promotes antisense transcription and formation of dsRNA. As a result, RNAi machinery and associated histone modification machinery, including G9a, localize at the pause site. Subsequent deposition of the repressive H3K9me2 mark on chromatin by G9a leads to recruitment of heterochromatin protein 1γ (HP1γ), which reinforces the pausing. Transcriptional termination subsequently occurs and prevents run-through at regions of high gene density to maintain gene integrity.

## MECHANISMS OF STRESS INDUCTION VIA R-LOOP ACCUMULATION

Accumulation of R-loops often results in increased cellular stress that leads to genomic instability. The current hypothesis states that stabilized, co-transcriptional R-loops disrupt RF progression during S-phase. This disruption causes stalling that can lead to fork collapse, DSBs, or incomplete replication before entry into mitosis. Recently, perturbation of the mRNA splicing, processing, and export factors have been shown to increase R-loop mediated genome instability. For example, disruption of the THO and Transcription Export (TREX) complex (discussed above) leads to a hyper-recombination phenotype in yeast and human cells [43, 44]. Interestingly, these mutants show genome-wide increases in histone H3 serine 10 phosphorylation (H3S10P) to promote chromatin condensation during mitosis. Evidence suggests that this modification, along with R-loop associated H3K9me2 deposition, impairs replication and leads to stress [45]. Independent studies demonstrate that dysfunction of the SRSF1 splicing complex increases R-loop formation detrimental to genomic stability [16]. In these cases, the observed phenotypes can be rescued through overexpression of RNase H, indicating the importance of resolving DNA-RNA hybrids to prevent DSBs or recombination. Resolving topological stress remains an important aspect of genome maintenance where accumulation of R-loops after TOP1 inhibition increases genomic rearrangements [46]. These events result from replication fork collisions with a transcription bubble that was likely paused by the formation of an R-loop.

The deposition of R-loops at promoter and terminator regions has been shown to regulate initiation, pausing, and termination of transcription. Perturbation of these regulatory events could lead to inappropriate changes in gene expression or genetic stress. Studies indicate that mutation of the THO complex impairs elongation, possibly through the formation of R-loop intermediates that prevent RNA Pol II processivity [47]. Moreover, these impairments facilitate the hyper-recombination effects described above. A second form of genetic stress occurs from the exposed ssDNA segment in the R-loop. This feature is susceptible to alterations via DNA-modifying or repair enzymes, as well as chemical mutagens [48]. AID activity or other factors may inappropriately target the ssDNA strand of the R-loop and create lesions in the genome that lead to point mutations or other forms of deleterious repair. Unsurprisingly, stabilization of co-transcriptional R-loops can localize mutagenesis to gene regions and exacerbate their effect by targeting coding sequence [44].

**Figure 2:**
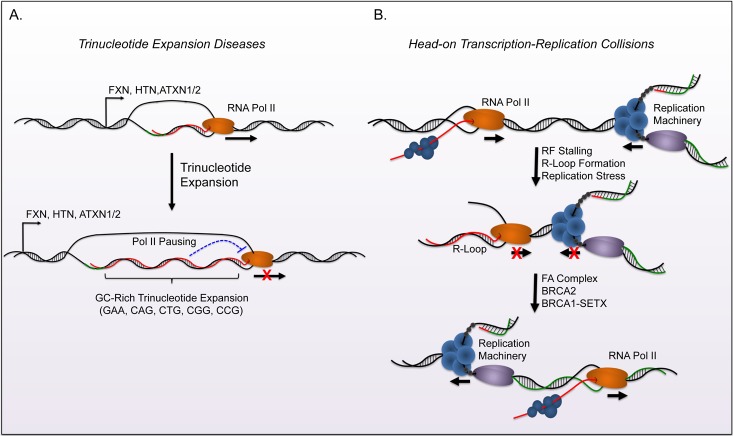
FIGURE 2: Biological consequences of R-loop formation. Formation of R-loops can result in defects in transcription and genomic instability. **(A)** Evidence indicates that R-loop formation contributes to trinucleotide expansion related diseases. The expansion of GC rich sequence in the gene body creates conditions that are favorable for R-loops and can subsequently cause reduced expression of specific genes (FXN, HTN, ATXN1/2). These diseases typically result in neurodegenerative disorders and ataxia. **(B)** Head-on collisions between the replication fork and transcription bubble create favorable conditions for R-loop formation. The FA complex, along with BRCA1, and other factors promote bypass and resolve R-loops during these collisions in order to complete replication and maintain genome stability. Mutation in FA complex subunits increase DNA damage associated with collisions and this damage is associated with cancer progression.

DNA damage sensing and repair proteins have recently emerged as inhibitors of R-loop accumulation in eukaryotic cells (**Figure 2**). For example, the Breast Cancer Type 1 and 2 susceptibility genes (BRCA1/2) prevent potential harmful effects of R-loops through two distinct manners. BRCA1 recruits the helicase SETX to R-loops that form at transcription termination pause sites or sites of Negative Elongation Factor (NELF) mediated Pol-II pausing [49, 50]. BRCA1-dependent recruitment of SETX resolves R-loop structures at these sites and suppresses DNA damage. Interestingly, studies of breast cancer models with a BRCA1 mutation show increased insertion-deletion (in-del) mutations at BRCA1-bound termination sites known to inhabit R-loops.

Depletion of BRCA2 from cells also increases R-loop accumulation, suggesting an important role for this protein in R-loop homeostasis. However, dissecting the specific role (or roles) of BRCA2 in resolving R-loops has been difficult due to its contributions in DNA repair, RF stability, and other mechanisms associated with mitosis. Its inherent involvement in these processes may alone prevent R-loop formation by preventing RF collapse and recruiting the ssDNA binding protein, Rad51, to DSB sites [51]. However, recent evidence shows that BRCA2 associates with the TREX complex subunit, PCI-domain containing protein 2 (PCID2), which suggests a protective role for BRCA2 against the co-transcriptional formation of R-loops [33]. Separately, research shows that BRCA2 recruits RNA polymerase II associated factor-1 (PAF1) to promoter-bound Pol-II, potentiates pause release, and decreases R-loop formation [52].

Both BRCA1 (FANCS) and BRCA2 (FANCD1) are part of the Fanconi Anemia (FA) mediated DSB repair pathway. Therefore investigators elucidated that this complex may regulate genome stability through R-loop resolution. In addition to the protective functions of BRCA1 and BRCA2, the disruption of critical FA complex members FANCD2, FANCA, and FANCM lead to genomic instability and DNA damage from R-loop mediated RF collapse [53, 54]. The activity of the FA complex during replication allows RFs to traverse intrastrand crosslinks (ICL), and promotes post-replicative repair of this lesion [55]. Briefly, the FA complex is recruited by ATR and subsequently activates FANCD2 through a mono-ubiquitination step. The complex then utilizes the DNA translocase activity of FANCM to traverse the lesion and prevent RF collapse. Emerging evidence demonstrates that the same FA complex mediated mechanisms facilitate RF progression through transcription-mediated conflicts, including R-loops. The FA complex is recruited to the stalled RFs due to collisions with the transcription machinery. It subsequently removes R-loops and restarts replication to prevent RF collapse. RNase H1 over-expression rescues the phenotypes observed during disruption of FA function and validates the role of R-loops in these events. Conversely, defects in the FA complex lead to increased R-loop formation upon treatment of ICL-inducing agents. This suggests that R-loops may be an outcome of genotoxic stress in FA patients. Interestingly, depletion of BRCA1 or BRCA2 individually shows that each gene regulates R-loop formation in distinct regions of the genome. Sequence or epigenetic differences that determine recruitment of BRCA1 or BRCA2 to R-loops remain an area of keen interest in the field.

The induction of stress due to R-loop accumulation largely results from collisions that occur between RFs and the transcriptional machinery, particularly in a head-on orientation [56]. Genomic instability and genome rear-rangements occur if these structures cannot be resolved through the replication stress responses of the FA complex, DNA repair, and R-loop homeostasis. This creates a positive feedback model where genomic instability can potentiate additional R-loop accumulation. Buildup of R-loops during stress may identify new mechanisms that contribute to the pathology of these structures.

## BIOLOGICAL OUTCOMES OF INAPPROPRIATE R-LOOP HOMEOSTASIS

R-loops can arise from a variety of cellular stresses, and lead to complications including replication defects, transcriptional irregularities, and genomic instability. The biological consequences of disrupted R-loop homeostasis has been described in multiple neurodegenerative diseases that largely associate with ataxia (Reviewed in [57]). GCrich trinucleotide expansions of gene regions provide an extended landscape for R-loop formation that disrupt transcription and proper gene expression (**Figure 2A**). For example, Huntingtin (HTT; Huntington's Disease), Frataxin (FXN; Friedreich Ataxia), and Ataxin 1/2 (ATXN1/ATXN2; Spinocerebellar Ataxias) all have GC-rich trinucleotide expansion tracks that form R-loops *in vitro* and associate with disease progression [58, 59].

R-loop induced disruption of gene expression occurs through distinct mechanisms in trinucleotide repeat disorders. Expansion of the repeat region in the first intron of FXN leads to R-loop formation, increased histone H3K9 dimethyl (H3K9me2) chromatin deposition, and impeded Pol II mediated transcription [60]. Chemical inhibition of TOP1 in Friedreich Ataxia (FRDA) model cell lines with FXN expansions potentiates R-loop accumulation and H3K9me2 enrichment, providing further evidence that R-loop formation increases deposition of this repressive mark.

Fragile X syndrome harbors a mechanism where trinucleotide expansion in the 5' UTR of the FMR1 gene leads to DNA methylation mediated silencing of this locus [60, 61]. However, removal of DNA methylation from this locus reactivates expression to only twenty-five percent of wildtype cells. The reactivation of FMR1 expression is partial because of R-loop formation after depletion of DNA methylation. Examination of the relationship between DNA methylation and R-loop formation at this locus could reveal a distinct mechanism of gene silencing in this model. Separately, trinucleotide repeat expansions can form R-loops that induce RNA Pol II pausing and aborted transcripts, which subsequently sequester nucleolin (NCL) and cause nucleolar stress [62, 63].

Furthermore, mutations in the machinery that maintain R-loop homeostasis also associate with neurodegenerative disease. Dysfunctional TREX or RNaseH2 are linked to Aicardi-Goutières Syndrome (AGS) while SETX mutations contribute to ataxia with oculomotor apraxia (AOA2) [64, 65]. Mutations of SETX also occur in the motor neuron disease amyotrophic lateral sclerosis 4 (ALS4). In this case, the SETX mutation decreases R-loops in the promoter region of BMP and activin membrane bound inhibitor (BAM-BI). Local R-loop loss increases DNMT-1 dependent methylation at the promoter, and downregulation of BAMBI leads to the activation of the TGF-β pathway, a process implicated in ALS [66].

As expected, the increased genome instability, chromosomal rearrangements, and DNA damage that occur during aberrant accumulation of R-loops is linked to oncogenesis [53, 67, 68] (**Figure 2B**). Loss of BRCA1 and BRCA2 function has already been well established in breast cancer. Cells show an increase in DSBs when lacking the protective effects that these genes carry against RF collapse [33, 49, 50, 69]. Moreover, the increased frequencies of in-dels in BRCA mutant cell lines localize these mutations to gene regions and can have deleterious effects on gene expression. The cancer-associated genotoxic stress that arises from mutations in BRCA1/2 or members of the FA complex can all be partially rescued through overexpression of RNase H1 in cancer cell lines, suggesting that the aberrant R-loop formation contributes to malignant progression.

The Tudor containing protein 3 (TDRD3) plays a critical role in maintaining proper R-loop homeostasis at the c-MYC locus [15]. TDRD3 recruits TOP3B to relieve negative super-coiling at c-MYC and prevents R-loop accumulation. Loss of TDRD3 function in B-lymphocytes leads genomic rearrangements between c-MYC and the Ig heavy chain (IGH) locus due to AID activity on R-loop associated ssDNA.

This translocation commonly occurs in Burkitt's lymphoma and multiple myeloma and causes oncogenic levels of c-MYC expression. Loss-of-function in mRNP biogenesis machinery also associates with known cancers. For example, sequestration of the THO/TREX complex through Kaposi's sarcoma herpesvirus expression of ORF57 leads to increased genomic instability and subsequent malignant progression in immunodeficiency-associated models [70].

In *Saccharomyces cerevisiae*, the formation of R-loops at the noncoding telomere repeat-containing RNA (TERRA) locus facilitates type II survival in telomerase mutants [71]. The survival mechanism depends on radiation sensitive (*RAD52*) dependent recombination events that result in long heterogenous tracts of telomeric repeats. Extended tracts of recombination dependent repeats appear in mammalian cells utilizing the alternative lengthening of telomere (ALT) pathway during bypass of senescence. Therefore, accumulation of R-loops at TERRA may also play a role in promoting ALT activation in some types of cancer.

Together, these models provide a wide variety of mechanisms where stress associates with increased R-loop formation and can lead to progressive neurodegenerative disease or cancer. The diversity of these mechanisms lends credence to the importance of proper R-loop homeostasis. Redundancy in the machinery that resolves R-loops suggests these structures are dynamic and widespread (studies indicate that R-loops occupy up to 5% of the genome [25]). As we increase our understanding of the formation and resolution of these lesions *in vivo*, we may discover new disease associations of R-loops.

## CONCLUDING REMARKS

Emerging evidence demonstrates the importance of proper R-loop homeostasis. These structures provide critical regulatory functions for gene expression and, in some cases, gene rearrangements. However, their formation and resolution must be tightly regulated to prevent topological events that disrupt replication through impaired RF progression. Such impairment provides an avenue for genome instability and contributes to the development of progressive diseases like cancer. Moreover, prolonged R-loop occupancy exposes ssDNA to genotoxic stress, which produces deleterious effects on gene expression.

Future examination of R-loops in cancer models should determine whether the seemingly independent functions of BRCA1 and BRCA2 are coordinated to prevent RF collapse through FANC-complex-mediated mechanisms. Specifically, research should elucidate whether BRCA1 recruits SETX to unwind R-loops while BRCA2 acts to traverse the lesion in a manner to complete replication and allow DNA repair to occur at a later phase. Moreover, delineating the coordination between RNase H, helicases, and topoisomerases to prevent R-loop accumulation could provide insight into why specific cell types are susceptible to diseases associated with improper R-loop homeostasis.

Finally, the link between the epigenetics and sites of R-loop formation has been established, but further examination of the machinery that recruits chromatin-modifying enzymes is warranted. Discovery of mechanisms that link R-loop homeostasis to chromatin modification would lend further clues into the development of specific cancers or neurodegenerative diseases. Specifically, the field would benefit from identification of R-loop “readers” that allow cells to detect these structures and modify chromatin to repress gene expression or promote transcriptional termination.

R-loops provide an interesting new source of replicative and transcriptional stress that clearly have disease-causing consequences. As new methodologies and models are developed to study their regulation and homeostasis, researches will continue to integrate this under-studied structure into cell biology and pathogenesis.

## Abbreviations:

AID: activation-induced deaminase,
ds: double-stranded,
DSB: double strand break,
FA: Fanconi Anemia,
G-cluster: guanine-rich cluster,
mRNP: messenger ribonucleoprotein particle,
RF: replication fork,
ss: single-stranded,
TOP: topoisomerase,
TREX: THO and transcription export.
